# The Memory Metal Minimal Access Cage: A New Concept in Lumbar Interbody Fusion—A Prospective, Noncomparative Study to Evaluate the Safety and Performance

**DOI:** 10.1155/2012/898606

**Published:** 2012-04-08

**Authors:** D. Kok, R. D. Donk, F. H. Wapstra, A. G. Veldhuizen

**Affiliations:** ^1^Department of Orthopedics, Universitair Medisch Centrum Groningen, Hanzeplein 1, Postbus 30.001, 9700 RB Groningen, The Netherlands; ^2^Department of Orthopedics, Canisius Wilhelmina Ziekenhuis Nijmegen, Weg door Jonkerbos 100, Postbus 9015, 6500 GS Nijmegen, The Netherlands

## Abstract

*Study Design/Objective*. A single-centre, prospective, non-comparative study of 25 patients to evaluate the performance and safety of the Memory Metal Minimal Access Cage (MAC) in Lumbar Interbody Fusion. *Summary of Background Data*. Interbody fusion cages in general are designed to withstand high axial loads and in the meantime to allow ingrowth of new bone for bony fusion. In many cages the contact area with the endplate is rather large leaving a relatively small contact area for the bone graft with the adjacent host bone. MAC is constructed from the memory metal Nitinol and builds on the concept of sufficient axial support in combination with a large contact area of the graft facilitating bony ingrowth and ease in minimal access implantation due to its high deformability. *Methods*. Twenty five subjects with a primary diagnosis of disabling back and radicular leg pain from a single level degenerative lumbar disc underwent an interbody fusion using MAC and pedicle screws. Clinical performance was evaluated prospectively over 2 years using the Oswestry Disability Index (ODI), Short Form 36 questionnaire (SF-36) and pain visual analogue scale (VAS) scores. The interbody fusion status was assessed using conventional radiographs and CT scan. Safety of the device was studied by registration of intra- and post-operative adverse effects. *Results*. Clinical performance improved significantly (*P* < .0018), CT scan confirmed solid fusion in all 25 patients at two year follow-up. In two patients migration of the cage occurred, which was resolved uneventfully by placing a larger size at the subsequent revision. *Conclusions*. We conclude that the Memory Metal Minimal Access Cage (MAC) resulted in 100% solid fusions in 2 years and proved to be safe, although two patients required revision surgery in order to achieve solid fusion.

## 1. Introduction

Chronic low back pain is an insidious problem. Individuals suffer from prolonged discomfort, anxiety, and disability. Low back pain has been shown as the leading cause of man-hours lost to disease or injury. Degeneration of the intervertebral disc is the most common cause of low back pain [1].

Conservative treatment for low back pain may include rest, heat, physical therapy, medication, bracing, and education. Most individuals will find relief given conservative treatments. However, for those with significant continuing specific symptoms, surgical intervention may be appropriate. One of the interventions is posterior lumbar interbody fusion (PLIF). The goal of spinal fusion is to obtain a solid arthrodesis. There is a wide range of fusion rates (56–95%) reported after PLIF with varying techniques [2–14].

The PLIF procedure was introduced independently by Jaslow [15] and Cloward [2, 16–18] in the 1940s to treat painful intervertebral disc damaged by degeneration or herniation. A PLIF has the advantages over other types of fusion allowing neural decompression while in the meantime restoration of the disc height, and segmental alignment is maintained [19]. 

In order to eventually achieve a solid interbody fusion a bone substitute has to be applied to the disc space. Without a mechanical support, these grafts tend to collapse, displace, or extrude [20–22]. For this reason, various metal and carbon fibre interbody cages have been developed [3, 23, 24]. Interbody fusion cages aim to fulfil both mechanical and biological requirements for fusion, in that the cages are designed to withstand high axial loads [19, 25, 26], and in the meantime to allow ingrowth of vital host bone. Although cages have rapidly become popular, the mismatch in the modulus of elasticity between many available metal cages and the actual vertebral body may cause stress shielding, resulting in a delayed fusion and eventually pseudarthrosis [27, 28]. Carbon fiber cages better approximate this modulus of elasticity of the vertebral bone; however, there are some reports on carbon fiber release causing synovitis [29]. The titanium implants developed by Kuslich et al. [23] and Ray [24] offer a radio-opaque alternative to carbon fibre materials that also exhibit the necessary biomechanical strength as well as facilitating the cage to be located radiographically. Their open design means that the bone is exposed to a greater graft surface area that has been shown to facilitate good bony in growth. However, the problem with most cages is the small contact area of the bone graft and, therefore, a high rate of pseudoarthrosis.

**Figure 1:**
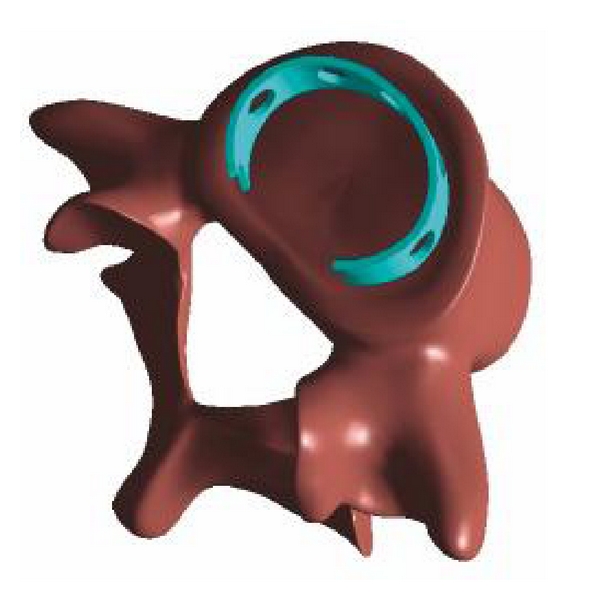
Memory Metal Minimal Access Cage (MAC).

**Figure 2:**
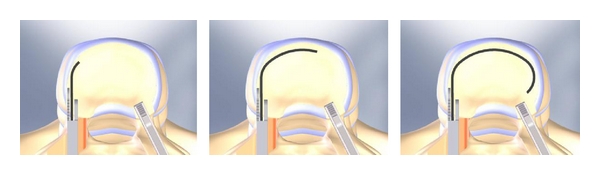
Implantation technique.

The Memory Metal Minimal Access Cage (MAC) builds on the concept of sufficient axial support in combination with a large contact area of the graft facilitating bony ingrowth and ease in minimal access implantation due to its high deformability. The MAC cage is a horseshoe-shaped implant. It confers the ability for fast and solid fusion due to the large contact area. The MAC cage is constructed from the memory metal nitinol (Figure 1). This device has the same modulus of elasticity as the vertebral body [30], allows a large bone surface contact area from the graft, and its high deformability will facilitate less invasive implantation in the future (Figure 2). Earlier biomechanical testing revealed an adequate subsidence resistance in human lumbar spine, comparable to or even better than the Harms cage [30]. The use of memory metals and their biocompatibility has already been described in earlier medical applications [31], as are the safety considerations [32].

The purpose of this pilot study was to evaluate the performance and safety of this new interbody fusion device in a relatively small group of patients.

## 2. Materials and Methods

### 2.1. Patients

Twenty-five consecutive patients (11 male and 14 female) with a diagnosis of a symptomatic single level degenerative lumbar disc consented to participate in the study, following Research Ethics Committee approval. The average age of the patients was 41.3 (range 23.8–71.4) Inclusion criteria required all patients aged 18 years and over, with disabling back and/or refractory radicular pain who have had at least six weeks of conservative management, with moderate-to-severe degenerative changes in one or two lumbar disc levels based on MRI performed not more than three months prior to study entry. In addition, discography had been provocative for patients back pain. Exclusion criteria ruled out patients with more than two abnormal lumbar disc levels, evidence of infection in the disc or spine, spinal tumor(s), who are immunocompromised, pregnant, and/or have a condition which would compromised their participation and followup in this study. Conservative treatment mostly entailed a combination of appropriate analgesics, physical therapy, and epidural and/or facet injections.

### 2.2. Implant Features and Surgical Procedure

The Memory Metal Minimal Access Cage has a horseshoe shape and comprises a material strip of 1.08 mm thickness for the small sizes, and 1.25 mm for the medium and large sizes. All cages have diamond-shaped holes for bone through growth, spikes on the top and bottom edges for stability, and a wedged profile. The diamond-shaped hole design aspect of the MAC is in line with surgical titanium mesh for similar product appearance, and seats on the bony outer cortical rim of the vertebral body.

The cages are made of nitinol, a shape memory alloy, which enables the surgeon to un-curve the strip completely, put it into an inserter, and insert it into the disc space while pushing it out of the inserter. The flat strip will henceforth curve into the original horseshoe shape (Figure 2). All surgeries were performed by two experienced spine surgeons between January 2004 and Oktober 2006. A standard PLIF procedure was performed using the Monarch TM pedicle screw system (DePuy International) where after the MAC cage was placed anteriorly in the intervertebral disc space (Figure 2), and locally available decompressive autologous bone was subsequently grafted into the disc space.

### 2.3. Clinical and Radiological Outcome

Patients were evaluated preoperatively at 1, 3, 6, 12, and 24 month after surgery. Evaluation at each interval included physical and neurological examination, concomitant medication, additional surgical procedures, subject completed questionnaires (Oswestry Disability Index, Short Form-36 Health), and Visual Analogue Scale for Pain. Any adverse events and complications were recorded in the case report forms.

**Figure 3:**
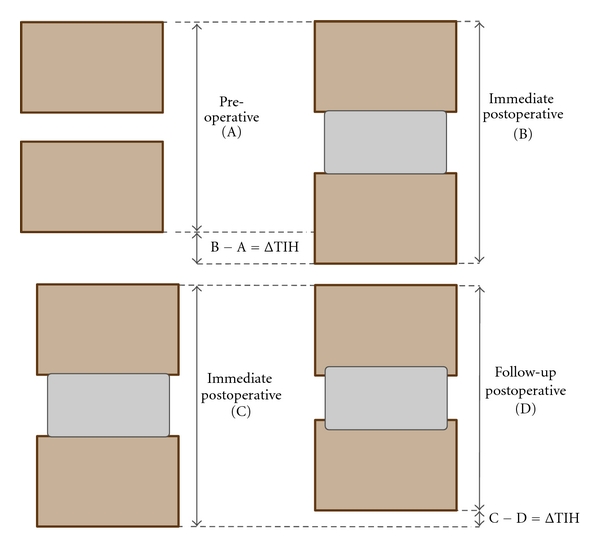
Measurement of the subsidence and total intervertebral height.

Routine lateral and AP radiographs were obtained at each timer interval. Routine radiographs were used to evaluate the total intervertebral height and subsidence. The CT scan at two years followup was used to establish fusion. The total intervertebral height (TIH) of two fused vertebral bodies was measured as distance between the mid-point of upper end plate of cranial vertebral body and the mid-point of lower end plate of caudal vertebral body on digital radiographs with built-in software (PACS viewer). The degree of subsidence (ΔTIH) was reflected by the difference between the immediate postoperative and follow-up TIH (Figure 3). With the same method, change of postoperative disc space height was reflected by the difference between TIH of the postoperative lateral plain radiograph and that of the preoperative lateral plain radiograph (Figure 3). Interbody fusion was defined as complete bridging at any one or more points within the central area of the vertebral body as determined by CT. Intervertebral fusion assessments were determined by one independent radiologist who was not otherwise involved in the study. Fusion was recorded as Yes/No/Can't Assess.

Complications were divided into device-related and non-device-related complications. Non-device-related complications were listed as major and minor.

### 2.4. Statistics

For statistical analysis, comparisons between pre- and postoperative scores were made using paired *t* tests.

## 3. Results

### 3.1. Radiological Assessment

The primary radiological objective was fusion rate. Fusion success was achieved in 25 (100%) of 25 patients. There was a solid bony fusion on CT at 2 years postoperative. The disc space height was restored to normal as part of the operative procedure. Disc height in the cage levels was increased from an average of 7.6 mm before surgery to an average of 12.4 mm after surgery losing 0.0 mm during healing in 2 years of followup.

### 3.2. Clinical Data

**Figure 4:**
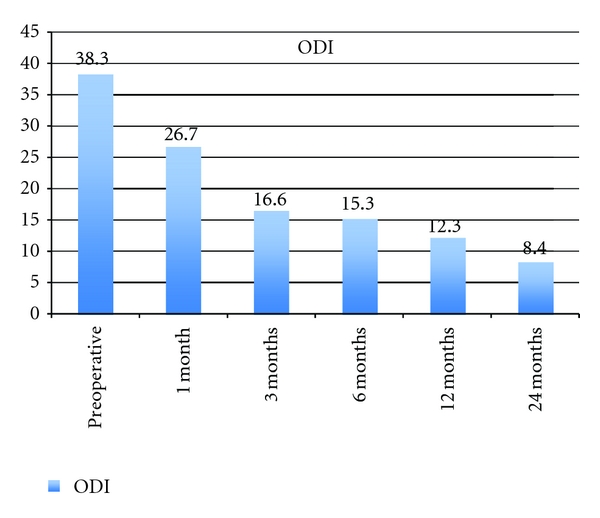
Oswestry Disability Index at baseline and 1, 3, 6, 12, and 24 months after operation.

**Figure 5:**
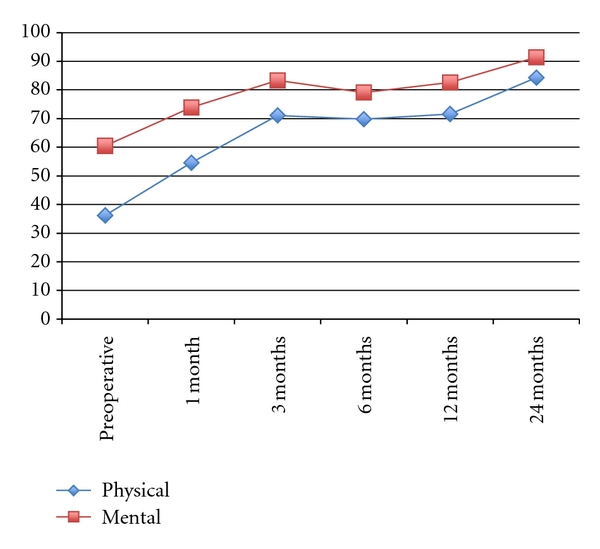
Short Form 36 (SF-36) Health Questionnaire (Physical and Mental) at baseline and 1, 3, 6, 12, and 24 months after operation.

**Figure 6:**
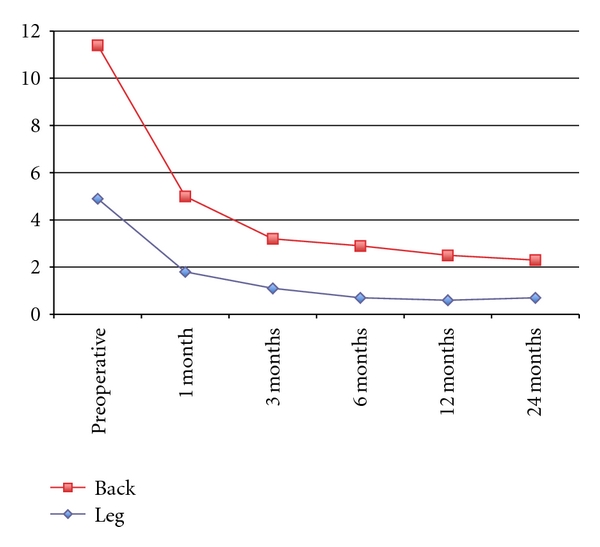
Pain Visual Analogue Scale (VAS) (Leg and Back) at Baseline and 1, 3, 6, 12, and 24 months after operation.

All 25 patients completed the 24 months of follow-up without any major adverse event. The clinical parameters are summarized Figures 4, 5, and 6.

The clinical outcome was the ODI score at 24 months posttreatment compared to baseline. The mean ODI score preoperative was 38.32 ± 10.64. This significantly improved to 8.4 ± 9.49 at 24 months postoperative (*P* < .0001).

 The Short-Form 36 health questionnaire (SF-36) data assessed both physical and mental components. Physical (PCM) 36.15 ± 18.93 improved to 84.25 ± 22.29 (*P* < .0001) and mental (MCM) 60.54 ± 24.22 improved to 91.36 ± 12.76 (*P* < .0001). Pain assessment (both leg and back) by Visual Analogue Scale (VAS) was also performed. Both leg and back pain improved significantly (*P* < .0001).

Bivariate analysis indicated that gender, previous nonsurgical treatment, smoking history, and obesity had no statistical effect on clinical or fusion success.

### 3.3. Safety

In two patients, an undersized implant was used, resulting in migration of the MAC cage, 1 day postoperatively, which required reoperation.

One patient had a myocardial infarction several days after surgery. There were no deaths or deep infections. There were 4 intraoperative dural penetrations in patients who had previous lumbar operations.

## 4. Discussion

In this study, a prospective followup on clinical and radiographic parameters was performed in patients with a single level spondylodesis using a new interbody cage design.

### 4.1. Radiological Assessment

Radiological assessment indicated that there was a 100 percent interbody fusion with the MAC device at 2 years on CT with no subsidence.

Previous studies [2, 4, 24, 33–38] report of PLIF fusion success with fusion in 85% of the cases. The difficulty in determining fusion success by standard roentgenographic methods was emphasized by Hibbs and Swift in 1929 [39], Cleveland et al. in 1948 [40], Prothero et al. in 1966 [41], Stauffer and Coventry in 1972 [42], Chow et al. in 1980 [43], Zinreich et al. in 1990 [44], and Brodsky et al. in 1991 [45]. The recent use of pedicle screw fixation has added to the problem, because overlying shadows of the implants impaired radiographic visualization of posterolateral fusion mass [13, 46]. Santos et al. in 2003 [47] emphasized that there is an overestimation of fusion on plain radiograph compared to CT.

In order to make a good estimation on interbody fusion, we used CT in this study. Previous studies on interbody fusion, reported significant loss of disc space height during healing of interbody grafts [2, 5, 22, 48–51]. In past reports, even pedicle screw stabilization has not prevented this loss of disc space height during the healing of interbody fusion. [4, 5, 48] Loss of disc space height creates foraminal narrowing and the potential for nerve root compression. The fact that we recorded 100 percent fusion on CT and no subsidence is an advantage over other interbody fusion devices.

### 4.2. Clinical Data

Numerous studies have provided subjective descriptions of criteria for excellent, good, fair, and poor results [36, 46, 52–57]. We use the ODI as our primary clinical objective because the ODI is valid and vigorous measure and has been a worthwhile outcome measure [58, 59].

The Oswestry Disability Index mean score preoperatively was 38.32 ± 10.64. This significantly improved to 8.4 ± 9.49 at 24 months postoperative. Significant improvement in both physical and emotional components in the SF-36 questionnaires mean scores was also observed, with increases from baseline results of 36.15 ± 18.93 and 60.54 ± 24.22 to 84.25 ± 22.29 and 91.36 ± 12.76 at 24 months, respectively (*P* < .0001). The average level of leg pain was reduced by more than 50% after operation (VAS values reduced from 4.88 ± 2.96 to 1.78 ± 1.97 at 1 month after operation). This reduction further improved over the 24 months after operation (0.73 ± 1.31 at 24 month after operation). A similar reduction in back pain was also revealed. With both ODI and SF-36 results, improvement in condition continued throughout the 24 months after operation. Pain results indicated a rapid improvement after operation, which was maintained during the 24 months after operation.

A study of 60 patients with posterior lumbar interbody fusion combined with instrumented posterolateral fusion reported by Freeman et al. [60] indicated stable circumferential fixation as shown by radiographs and tomograms confirming the presence of a bridging fusion mass. Of the 48 ODI questionnaires completed after 5 years, 79% had an ODI <30. In the present study, 96% (24/25) of the patients indicated an ODI < 30. McKenna et al. reported a prospective, randomized controlled trial of femoral ring allograft (FRA) versus a titanium cage (TC) in circumferential lumbar spinal fusion with minimum 2 years clinical results [61]. Comparison of change in ODI results indicated a significantly larger improvement in the FRA group (reduced from 57 to 42) when compared to the TC group (54 reduced to 48). The corresponding change in ODI results from baseline over 2 years in the current study was larger than that of either the FRA or TC groups (35 versus 15 and 6). SF-36 results for the FRA patients showed a significant improvement in the Physical Function Component but not in the Mental Component (change in SF-36 results of 17 and 2 resp.). In the TC patients, the reverse was found (change in SF-36 results of 5 and 9 for Physical Function and Mental Components, resp.). The MAC in comparison gave a much greater improvement in both SF-36 results (change in SF-36 results of 63.1 and 27 for Physical and Mental Components, resp.). Both FRA and TC patients showed a significant improvement in VAS for back pain (change in VAS 1.9 and 1.1, resp.). However, with leg pain VAS scores only FRA patients demonstrated a significant improvement (change in VAS of 1.3), whereas the TC group had more leg pain increasing the VAS scores postoperatively by 0.4 points. In our study, we found a significant reduction in both back and leg pain. With the MAC, the back and VAS results were reduced by 6.4 and 5.8 points, respectively. This indicates a significant improvement compared to the McKenna study. Cassinelli et al. published a prospective clinical study of revision fusion surgery in 19 patients with pseudoarthrosis who had received posterior lumber interbody fusion using stand-alone metallic cages [62]. SF-36 and ODI data were collected prior to surgery and two years postoperatively. Significant improvement was only noted in two of the eight SF-36 subcategories (Physical Functional and Role Mental). There was no significant difference in ODI scores. A study with two different patient groups of 30 subjects having spondylolisthesis which were subjected to different surgeries: posterior lumbar fusion with pedicle screws (Group I) and posterior lumbar interbody fusion with pedicle screws (Group II) has also been reported [63]. The ODI mean scores preoperatively and 2 years postoperatively were 28.5 and 18.6, respectively for, Group I and 31.3 and 13.3, respectively, for Group II. The ODI scores in the current study show a greater improvement. Glassman et al. reviewed the ODI and SF-36 outcomes in a multicentre lumbar fusion study with followup after 2 years [64]. The minimal clinically important difference (MCID) seeks to differentiate a magnitude of change, which is not only statistically valid but also of real clinical value. Figures for MCID for ODI results have been reported as low as a 4-point decrease [65] and also a 10-point decrease [65]. The Food and Drug Administration (FDA) standards suggest a 15-point decrease in ODI and either maintenance of or any improvement in SF-36 Physical Composite Score (PCS) [66]. Ware et al. [67] reported that an increase of 5.42 points in the SF-36 PCS is clinically important. A more recent study [68] has reported the following MCID values: 12.8 points for ODI, 4.9 points for SF-36 PCS, 1.2 points for back pain, and 1.6 points for leg pain. The improvement in ODI values for the various fusion treatments in the multicentre review ranged from 9.9 to 22.2 points, whereas the improvement in SF-36 data ranged from 13.8 to 6.3 points. The improvement in the corresponding ODI and SF-36 values in the current MAC study were 29.92 and 39.46. The improvement in back and leg pain were 4.88 and 4.15, respectively. In general, the ODI and VAS improved in all PLIF-procedures, according to the literature. The results obtained for the MAC have, therefore, satisfied the MCID reported in the literature.

### 4.3. Safety

The device-related adverse event recorded in this study was two undersized cages, resulting in migration.

The migration problem lies within the operation technique.

The dural penetrations all developed during decompression in patients who were previously operated on, not during cage insertion, and were repaired at surgery, not requiring reoperation, not causing neurologic injury, and not affecting the hospital course.

## 5. Conclusion

The Memory Metal Minimal Access Cage performed very well radiographically and clinically. There was a 100 percent interbody fusion at 2 years on CT, no subsidence and significant improvement of clinically important outcomes, although two patients required revision surgery in order to achieve solid fusion.
